# The role of point-of-care ultrasound in the evaluation and management of hyponatremia: a systematic review

**DOI:** 10.1007/s11739-025-04256-z

**Published:** 2026-01-13

**Authors:** Francisco Javier del Castillo Tirado, Laisa Socorro Briongos Figuero, Samuel García-Rubio, Yale Tung-Chen, Luis Matías Beltrán Romero

**Affiliations:** 1 Department of Internal Medicine, Hospital de Santa Bárbara de Puertollano, Puertollano, Spain; 2 Department of Internal Medicine, Hospital Santos Reyes, Aranda del Duero, Spain; 3 Department of Medicine, Dermatology and Toxicology, Faculty of Medicine, University of Valladolid, Valladolid, Spain; 4 Department of Internal Medicine, Hospital Marqués de Valdecilla, Santander, Spain; 5 Department of Internal Medicine, Hospital Universitario La Paz, Paseo Castellana 241, 280246 Madrid, Spain; 6 Department of Medicine, Universidad Autónoma de Madrid, Calle Arzobispo Morcillo 4, 28029 Madrid, Spain; 7 Department of Internal Medicine, Hospital Universitario Virgen del Rocío, Seville, Spain; 8 Department of Medicine, Facultad de Medicina, Universidad de Sevilla, 41009 Seville, Spain

**Keywords:** Hyponatremia, Point-of-care ultrasound, Ultrasonography, Hypervolemia, Hypovolemia

## Abstract

Hyponatremia is the most common electrolyte disorder. Accurate assessment of extracellular fluid volume status (hypovolemic, euvolemic, or hypervolemic) is essential for determining the underlying etiology and guiding treatment. Point-of-care ultrasound (PoCUS) has emerged as a complementary bedside tool to objectively assess volume status in hyponatremia. We systematically reviewed the relevant literature up to June 2025. The aim of this systematic review was to evaluate the role of PoCUS in the assessment and management of hyponatremia. The evidence suggests that combined lung, cardiac, and abdominal PoCUS (including the venous excess ultrasound score, VExUS) helps overcome the limitations of physical examination. PoCUS leads to a more accurate etiologic diagnosis and optimized treatment. PoCUS also aids in monitoring treatment response, enabling real-time adjustment of diuretics or fluids based on serial examinations. Its role may be limited by operator dependency and inability to detect non-ultrasound-revealed pathologies such as hypothyroidism. Integrating bedside ultrasound into hyponatremia management improves volume assessment accuracy and supports a more individualized approach. Although current evidence is mainly derived from small studies and case series, findings consistently support using PoCUS as an adjunct to traditional evaluation.

## Introduction

Hyponatremia, defined as serum sodium concentration < 135 mEq/L, is the most common electrolyte disorder, affecting approximately 30–40% of hospitalized patients [1–3]. It is associated with increased morbidity and mortality [4]. Proper management hinges on determining the underlying cause, which is primarily inferred from extracellular fluid volume status:hypovolemia, euvolemia, or hypervolemia [5].

However, accurately classifying volume status is challenging. Physical examination and routine laboratory parameters are often unreliable. Even experienced clinicians misclassify volume status in approximately 50% of cases [6, 7]. For example, classical signs of hypovolemia (dry mucosa, orthostasis) or hypervolemia (edema, crackles, JVD) lack sufficient sensitivity and specificity [8, 9]. Furthermore, clinical scenarios like hypothyroidism or heart failure can obscure volume interpretation due to overlapping features [10, 11].

This diagnostic uncertainty can lead to inappropriate management. For instance, mistaking SIADH for hypovolemia might result in isotonic saline infusion, exacerbating the hyponatremia [12, 13]. Conversely, fluid restriction in true hypovolemia perpetuates sodium loss and high ADH levels [14].

Point-of-care ultrasound (PoCUS) has emerged as a useful adjunct to enhance bedside volume assessment. It allows real-time visualization of volume indicators (IVC diameter, pulmonary congestion, venous Doppler) that help clinicians infer the underlying volume status [15–18].

Despite its growing use in nephrology, internal medicine, and critical care [19, 20], no prior systematic review has consolidated the evidence base for PoCUS specifically in hyponatremia management. This review aims to fill that gap by synthesizing existing literature and outlining how PoCUS findings correlate with diagnosis, management decisions, and clinical outcomes in hyponatremia.

## Methods

Two reviewers independently screened titles and abstracts, and the full text of potentially eligible studies featuring a systematic literature review on the use of point-of-care ultrasound in hyponatremia. The search was limited to publications up to June 2025. We queried multiple databases (PubMed, ScienceDirect, TripDatabase, and Google Scholar) using MeSH terms and keywords in English and Spanish. Key search terms included “hyponatremia”, “hiponatremia”, “point-of-care ultrasound”, “POCUS”, “volume status assessment”, “VExUS”, “lung ultrasound”, “bedside ultrasound” and “SIADH”. The search was restricted to the last 10 years and to articles in English or Spanish.

Inclusion criteria comprised original studies (randomized trials, observational studies, and case series), clinical practice guidelines, and relevant narrative reviews that evaluated the use of PoCUS in adult patients with hyponatremia. Eligible studies had to evaluate point-of-care ultrasound (focused cardiac, lung, or IVC ultrasound performed at bedside) as a tool in the assessment or management of hyponatremia [21, 22]. Comparative studies with or without a control group (PoCUS vs. standard clinical assessment) were eligible, as well as observational studies without a comparison. We excluded studies focusing on individuals under 18 years of age, animal models, molecular or biochemical aspects without clinical application, and conference abstracts not published in peer-reviewed journals.

Titles and abstracts of retrieved articles were first screened for relevance to the review question. Publications deemed relevant were examined in full text to confirm eligibility and extract data. Extracted data included study characteristics (authors, year, country, design, clinical setting), patient demographics (number of patients, age range, etiology of hyponatremia if reported), details of the ultrasound intervention (what PoCUS exam was performed, who performed it, and when in the course of care), the reference standard or comparator (if any, with the inclusion of clinical exam or other tests), and all reported outcomes of interest. For diagnostic accuracy studies, we collected measures such as sensitivity, specificity, or accuracy of PoCUS [23, 24]; for management-focused studies, we noted changes in treatment decisions or time to correction of sodium attributed to PoCUS use, as well as any clinical outcomes (such as ICU admission, length of stay, mortality) reported.

Any discrepancies in data extraction between reviewers were resolved by consensus. Duplicates and studies of low applicability were removed. In total, 65 references were identified as the evidence base for this review. The findings were synthesized narratively and organized around key themes: (a) the importance and challenges of volume status assessment in hyponatremia; (b) the utility of PoCUS in the evaluation and follow-up of hyponatremic patients; (c) clinical evidence on the impact of PoCUS in hyponatremia management; and (d) the role of PoCUS in guiding therapy and monitoring outcomes.

### Risk of bias assessment

Two reviewers (FJCT and LMBR) independently assessed the risk of bias for each included study. For observational studies (including case series, cross-sectional, and cohort designs), we used an adapted version of the Newcastle–Ottawa Scale (NOS), which evaluates risk of bias across three domains: selection of participants, comparability of study groups, and ascertainment of outcomes [25]. Each study was categorized as having low, moderate, or high risk of bias based on the total score and the presence of critical limitations in any domain.

For case reports, which are inherently descriptive and lack comparator groups, we assessed methodological quality using criteria based on the CARE (CAse REport) guidelines, focusing on clarity of clinical presentation, diagnostic reasoning, intervention description, and outcome reporting [26]. Two narrative reviews with embedded case analysis and structured clinical reasoning (Berend et al. Koratala & Kazory) were appraised based on transparency, internal consistency, and clinical applicability.

For the single randomized controlled trial identified, we applied the Cochrane risk of bias 2.0 tool, assessing domains, such as randomization process, deviations from intended interventions, missing outcome data, measurement of the outcome, and selective reporting [27].

Discrepancies between reviewers were resolved by consensus. No third-party adjudication was necessary. The results of the risk of bias assessments are summarized in Table 1.

**Table 1:** Summary of study characteristics for included articles on PoCUS in hyponatremia, and result of the risk of bias assessment

Study (author, year)	Country/setting	Design	Sample size	Population	PoCUS intervention	Comparator	Main outcomes	Risk of bias tool	Risk of bias judgment
Martínez et al. [39]	Colombia/ICU and surgical ward	Case series	5	Hyponatremic patients (severe or symptomatic)	Multi-organ PoCUS (lung, heart, IVC, VExUS)	Clinical assessment	Change in diagnosis and therapy	NIH quality assessment tool for case series studies	Moderate
Evins and Rao [41]	USA/Hospital ward	Case report	1	Severe hyponatremia	Lung, IVC, pleural effusions	Clinical impression	Avoided inappropriate fluid therapy	CARE guidelines for case reports	Low
Chatterjee and Koratala [24]	USA/Hospital	Case report	1	Hyponatremia with occult HFpEF	Cardiac PoCUS (IVC, diastolic function)	Clinical evaluation	Detected hidden hypervolemia, diuresis	CARE guidelines for case reports	Low
Samant and Koratala [45]	USA/Nephrology service	Case report	1	Hyponatremia in cardio-renal syndrome	Repeated VExUS, Doppler veins	Diuretic response	Diuretic resistance, dialysis guided by PoCUS	CARE guidelines for case reports	Low
Rahman et al. [46]	UK/Inpatient nephrology	Prospective cohort	19	Severe hyponatremia (Na < 120 mEq/L)	Cardiac, lung, IVC PoCUS by nephrologist	Clinical diagnosis	Improved etiological accuracy of diagnosis	NIH quality assessment tool for observational cohort and cross-sectional studies	Moderate
Şirin et al. [47]	Turkey/Emergency department	Prospective cohort	110	Symptomatic hypotonic hyponatremia	IVC ultrasound only	Clinical diagnosis	IVC diameter correlation with status	NIH quality assessment tool for observational cohort and cross-sectional studies	Moderate
Bhardwaj et al. [23]	Indi/Surgical ward	Prospective cohort	107	Symptomatic hypotonic hyponatremia	Femoral Doppler	VExUS score, central venous pressure	Accuracy	NIH quality assessment tool for observational cohort and cross-sectional studies	Low
Marques et al. [38]	Spain/Internal medicine ward	Observational	26	Hyposmolar hyponatremia	VExUS and IVC measurements	Physical exam	Reclassification of volume status, no effect on Na	NIH quality assessment tool for observational cohort and cross-sectional studies	Moderate
Berend et al. [42]	Netherlands/Multicenter	Narrative framework + Diagnostic modeling	–	Mixed hyponatremic syndromes	Structured diagnostic algorithm (3-step) + web-based platform	None	Framework incorporating PoCUS to reduce diagnostic ambiguity	Not applicable (conceptual study)	Not applicable
Koratala and Kazory [43]	USA/Nephrology and education	Expert commentary	–	General nephrology context	Integration of PoCUS into nephrology workflow; training emphasis	None	Highlighted need for standardized PoCUS curricula and supervised competency	Not applicable (conceptual/educational study)	Not applicable
Vogt et al. [44]	Germany/Academic hospitals	Randomized controlled trial protocol	Planned *n* = 120	Thiazide-associated hyponatremia	Structured imaging parameters (lung, IVC, cardiac ultrasound) as co-variables	Standard diagnostic and therapeutic algorithm	Ultrasound parameters used to refine phenotypic classification, not primary endpoint	Cochrane risk of bias 2.0 (protocol-based)	Low (anticipated)

We did not perform a formal GRADE assessment of the certainty of evidence, as the outcomes and study designs were varied and primarily observational. However, we qualitatively discuss the evidence in the discussion section. No quantitative meta-analysis was performed due to heterogeneity in study designs and outcomes [28].

## Results

### Study selection and general characteristics

**Fig. 1:**
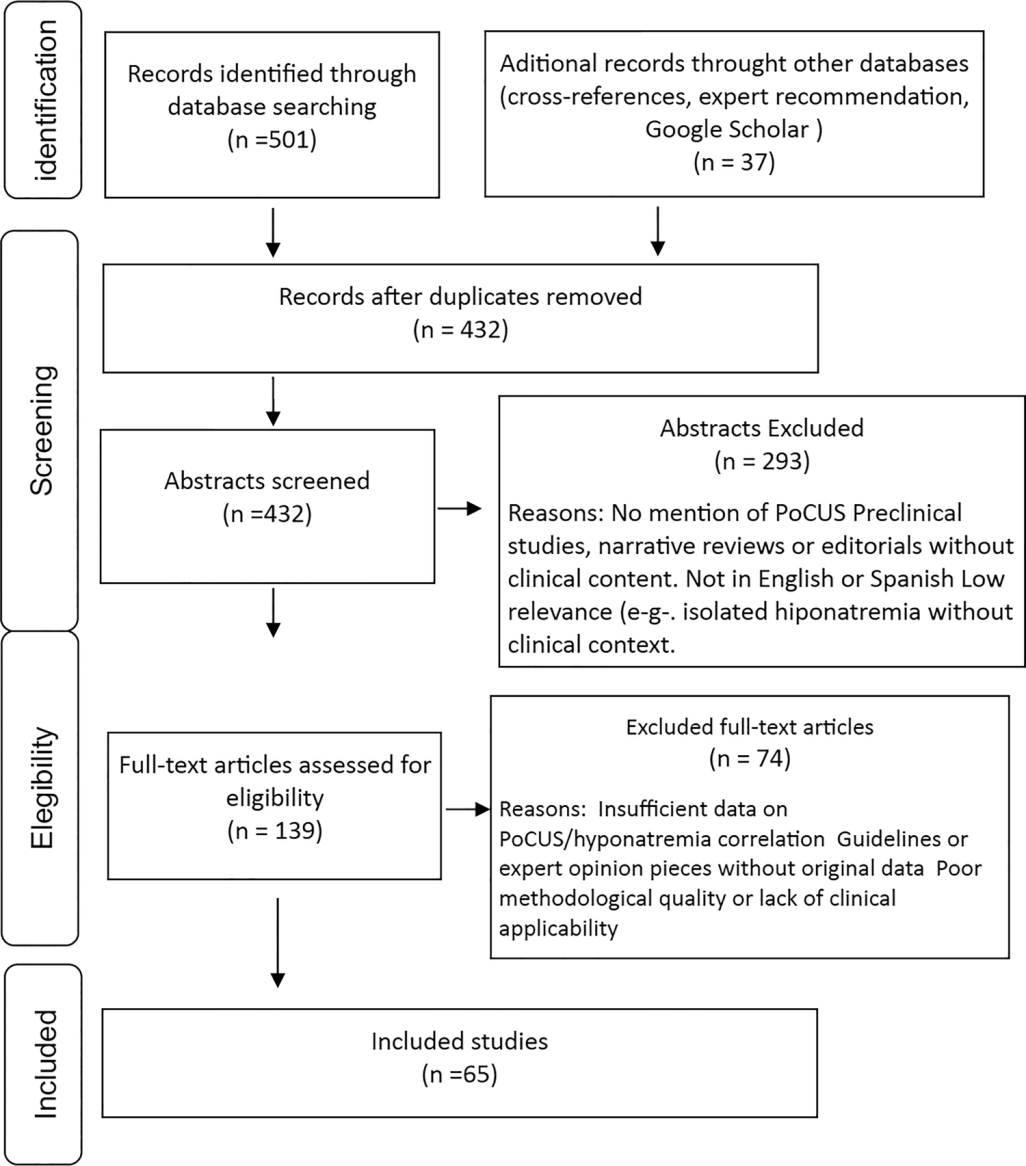
PRISMA flow diagram

**Fig. 2:**
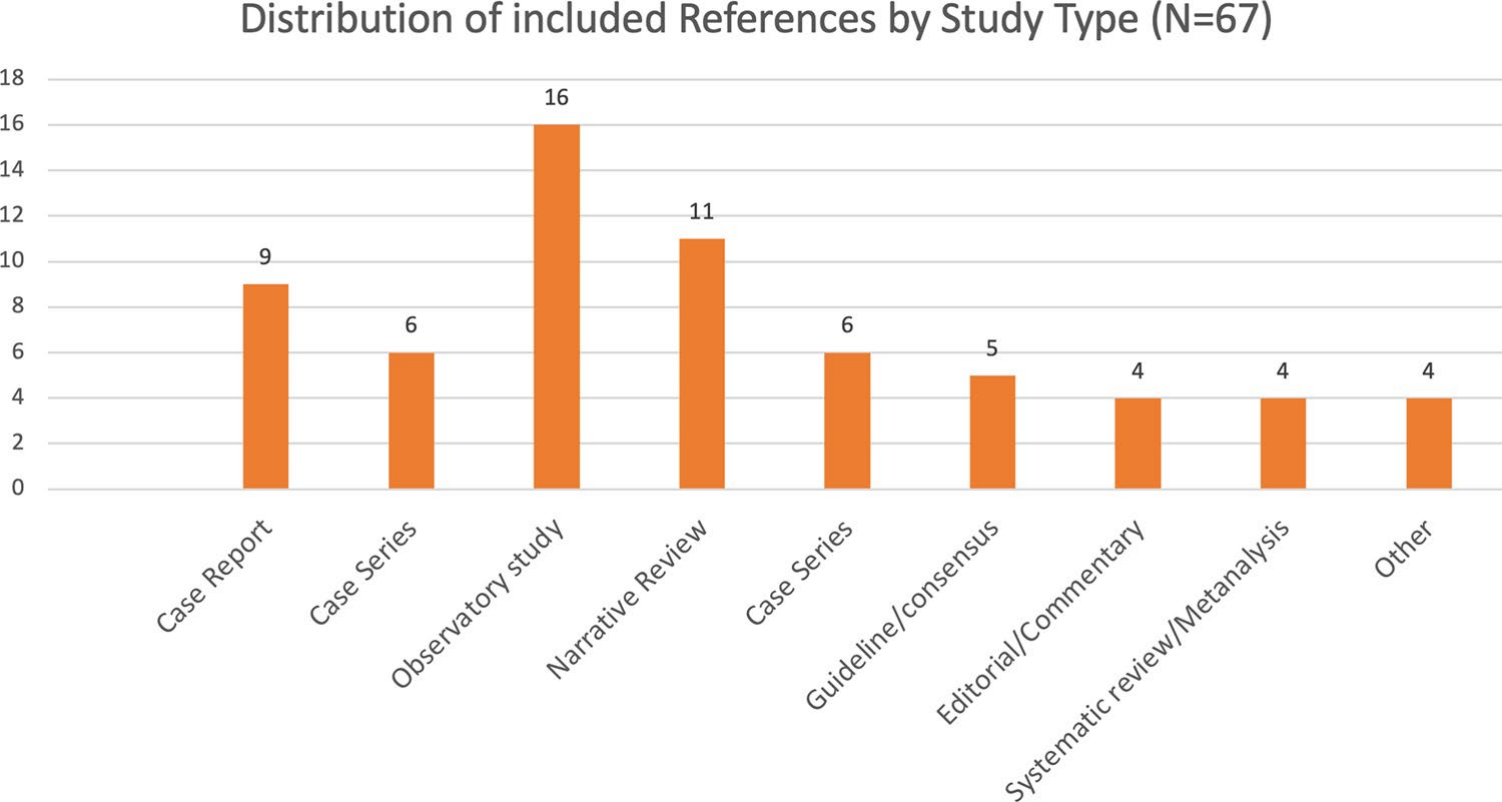
Distribution of included studies by design

A total of 11 studies met the inclusion criteria and were included in this systematic review [24, 34, 37–42]. The selection process is illustrated in the PRISMA flowchart (Fig. 1), and individual study characteristics are summarized in Table 1 and Fig. 2.

Of the 11 studies, 3 were single-patient case reports [24, 41, 42], 2 were case series [34, 39], 3were prospective cohort studies [37, 38], *1 was a narrative review with structured diagnostic framework*, *1 was a focused expert commentary with clinical case application*, and 1 was a randomized controlled trial [40]. Most studies were conducted in hospital-based settings—including intensive care, internal medicine, and nephrology departments—across Spain, Colombia, Turkey, the Netherlands, the United States, and India.

Sample sizes ranged from 1 (case reports) to 110 patients in the largest cohort study [38]. Some studies focused specifically on patients with severe hyponatremia (serum sodium < 125 mEq/L) [38, 39], while others included broader clinical presentations. The populations were heterogeneous, encompassing etiologies, such as SIADH, heart failure-related hypervolemia, and hypovolemia secondary to dehydration or diuretic use [4, 9].

All studies utilized point-of-care ultrasound (PoCUS) as part of the hyponatremia assessment. IVC ultrasound was used in all eleven studies, typically evaluating diameter and collapsibility [6, 7]. Lung ultrasound (for B lines) was used in 6 studies [24, 34, 39, 40, 43, 45], and focused cardiac ultrasound in 4 [24, 40, 43, 45]. On conceptual study addressed PoCUS as a diagnostic adjunct without reporting specific imaging parameters [42]. Five studies incorporated multi-organ PoCUS protocols [34, 38–40, 42, 43, 45], and five used the VExUS scoring system or a simplified equivalent [34, 38, 42, 43, 45].

Only two studies explicitly compared PoCUS-based assessments to standard clinical evaluation: a prospective cohort study by Marques et al. [38] and a randomized trial by Carrasco-Molina et al. [40], both of which showed that PoCUS improved diagnostic accuracy or confidence. *The remaining studies were descriptive, observational, or conceptual in nature but consistently reported that PoCUS findings influenced diagnostic reasoning or guided therapeutic decisions* [5, 20, 24, 34, 37, 39, 41, 43, 45].

### Limitations of clinical volume assessment

Clinical assessment of volume status in hyponatremic patients has significant limitations. In a classic study, only about 47% of hypovolemic and 48% of normovolemic hyponatremic patients were correctly identified by experienced physicians based on physical examination, meaning roughly half of cases were misclassified [6, 7]. Traditional diagnostic algorithms often rely on laboratory parameters—such as plasma osmolality to confirm true hypotonic hyponatremia, and urine osmolality and sodium to infer impaired free water excretion—but these indirect markers are often difficult to interpret and are not readily available at the bedside [5, 13]. *These limitations underscore the need for a multi-modal approach that combines clinical, laboratory, and imaging data, a view supported by structured diagnostic proposals that integrate PoCUS into clinical reasoning beyond isolated parameters* [20, 42].

Accordingly, there has been growing interest in PoCUS as a way to improve upon the inadequacies of the physical exam particularly in internal medicine and nephrology settings where training, adoption, and diagnostic reliability have been increasingly emphasized [6, 13, 19, 20, 43].

### Point-of-care ultrasound in initial volume evaluation

Bedside ultrasonography offers a rapid, multi-organ assessment of a patient’s volume status [12, 28]. The PoCUS approach in hyponatremia typically includes evaluation of the lungs, heart, inferior vena cava (IVC), and abdominal venous system (often summarized by the Venous Excess Ultrasound Score, VExUS) [9, 16, 23]. *Case-based and implementation-focused contributions support this multi-organ strategy, highlighting its applicability even outside intensive care settings* [43, 45]. Together, these ultrasound findings provide objective parameters of volume overload or depletion that complement clinical judgment [8, 37].

### Lung ultrasound

The presence of diffuse bilateral B lines on lung ultrasound is highly suggestive of interstitial pulmonary edema (congestion), whereas predominantly A lines (dry lung fields) indicate no significant pulmonary edema, consistent with a hypovolemic or euvolemic state [15, 28, 29]. Lung ultrasound is highly sensitive in detecting incipient pulmonary edema, often revealing congestion before crackles are audible [30].

### Cardiac ultrasound

*A focused cardiac ultrasound can assist in identifying structural and functional cardiac findings that, when integrated with clinical and sonographic data, may contribute to volume assessment in hyponatremic patients. Ventricular dilatation or left ventricular hypertrophy may be observed in heart failure, but these features alone do not confirm intravascular volume overload or elevated filling pressures *[31]*. This distinction is essential as structural abnormalities may reflect chronic remodeling rather than acute hemodynamic congestion *[24, 31, 33]*. Therefore, interpretation should incorporate complementary parameters, such as right ventricular size, septal motion, and inferior vena cava behavior, rather than relying on isolated structural features.*

*A more robust hemodynamic assessment of hypervolemia may include diastolic function grading, pulmonary artery systolic pressure estimation, and Doppler-based indices such as the E/e′ ratio. These parameters generally require additional training and are more often applied within comprehensive echocardiographic protocols. Estimation of stroke volume and cardiac output is also feasible with conventional echocardiographic methods, using LVOT measurements and Doppler recordings *[30, 43, 45]*.*

### Inferior vena cava

Measurement of the IVC diameter and its respiratory collapsibility is a classic PoCUS method to estimate intravascular volume. A small IVC that collapses > 50% with inspiration suggests low intravascular volume (hypovolemia), whereas a plethoric, minimally collapsible IVC indicates elevated central venous pressure (hypervolemia) [6, 7]. There are important exceptions: for example, patients with SIADH (euvolemic hyponatremia) may have a collapsible IVC despite normal volume status, while those with chronic pulmonary hypertension or with massive ascites can have a distended IVC despite effective volume depletion [9, 35]. Thus, IVC ultrasound findings must be interpreted in context and alongside other clinical and ultrasound clues to avoid misdirection [6, 7, 35].

### Venous congestion (VExUS) score

A more comprehensive assessment of venous congestion can be achieved with the Venous Excess Ultrasound Score (VExUS), which integrates IVC findings with Doppler evaluation of flows in the portal, hepatic, and renal veins [16, 23]. A high VExUS grade (II–III) reflects significant systemic venous congestion, whereas a low grade (0) indicates no major congestion. By examining multiple venous territories, VExUS can uncover occult volume overload that might be missed using the IVC alone [16, 23]. VExUS also correlates with invasively measured right atrial pressure to a moderate degree [37].

Studies have noted that PoCUS frequently changes the initial volume status classification. For example, clinicians have intensified diuretic therapy in patients who appeared euvolemic on exam but showed Doppler signs of congestion on ultrasound [13]. However, one observational study found that while PoCUS reclassified many patients, this did not significantly change the rate of serum sodium correction over 24–96 h [26]. On the other hand, two recent studies reported that higher VExUS scores were associated with greater difficulty in correcting hyponatremia and with diuretic resistance in acute cardiorenal syndrome [26, 31].

Overall, the VExUS approach remains promising for refining hyponatremia assessment—especially in complex cases—though further research is needed to validate its utility in broader patient populations [9, 16, 23, 26, 37].

### Clinical impact of PoCUS on management

Emerging evidence indicates that adding PoCUS to the evaluation of hyponatremia can directly influence management and improve diagnostic confidence [20, 37, 46].

### Case reports and series

Several case reports and case series illustrate how PoCUS findings can change clinical decision-making. Martínez et al. (2024) reported a series of ICU patients with hyponatremia in which multi-organ PoCUS led to revisions of the initial volume status assessment [39]. Notably, one case was reclassified from apparent hypervolemia to hypovolemia after PoCUS revealed a collapsed IVC and VExUS 0, prompting appropriate saline resuscitation and rapid normalization of serum sodium. Similarly, Chatterjee et al. used cardiac PoCUS to uncover occult heart failure with preserved EF in a hyponatremic patient, prompting diuresis [24]. Evins and Rao confirmed hypervolemia via PoCUS in a patient with severe hyponatremia and guided successful therapy [41]. Berend et al. [42] described a structured diagnostic algorithm integrating PoCUS into hyponatremia workup, which helped resolve conflicting volume indicators in challenging cases. Koratala and Kazory [43] emphasized the role of clinician training in avoiding misclassification and optimizing bedside ultrasound interpretation. Samant and Koratala reported persistent venous congestion in a hyponatremic patient with diuretic resistance, prompting initiation of ultrafiltration [45].

### Prospective studies

Rahman et al. prospectively evaluated 19 patients with severe hyponatremia (Na < 120 mEq/L) and reported that diagnostic concordance with the final etiology increased from 63% using clinical assessment alone to 84% when PoCUS was incorporated [46]. In this study, however, the final diagnosis was adjudicated retrospectively via chart review without an independent or blinded standard, introducing potential verification bias. Şirin et al. assessed IVC ultrasound in hyponatremic patients and classified volume status based on standard clinical and laboratory parameters alongside treatment response, without applying external reference criteria [47]. Both studies lacked a validated gold standard, which may have led to overestimation of PoCUS accuracy. Koratala likewise emphasized that isolated interpretation of IVC findings, without integration of broader clinical context or additional ultrasound views, risks diagnostic misclassification [35].

### Expert reviews

Recent narrative reviews support integrating PoCUS into hyponatremia workup [4, 5, 19, 20, 28]. Bhasin-Chhabra et al. highlight its utility in differentiating volume states. Mazón et al. propose a combined clinical-ultrasound diagnostic algorithm [20]. These authors, among others, advocate for applying individualized, PoCUS-guided management in hyponatremia [4, 19, 20].

### Role of PoCUS in monitoring and therapy

PoCUS enables serial monitoring of volume status and real-time therapy adjustment. In heart failure-related hyponatremia, lung ultrasound can be repeated to assess pulmonary congestion (via B lines), and IVC evaluation can help guide fluid removal or diuretic titration [5, 9, 12]. In one illustrative case, serial PoCUS showed resolution of B lines and normalization of IVC collapsibility after treatment, confirming effective decongestion [5].

In hypovolemic hyponatremia, repeat PoCUS may show persistence of a very collapsible IVC and hyperdynamic cardiac function, supporting further fluid administration [12]. Conversely, appearance of B lines and decreased IVC collapsibility may indicate fluid overload and suggest stopping resuscitation [12, 20].

In SIADH or chronic euvolemic hyponatremia, serial PoCUS helps confirm that fluid restriction is not inducing volume depletion. In cirrhosis, bedside ultrasound can estimate ascites and guide paracentesis [20, 39]. In cases with altered mental status, transcranial Doppler may suggest raised intracranial pressure, although still investigational [37].

Femoral vein Doppler is emerging as a simpler PoCUS tool for assessing venous congestion and shows promise as an alternative in limited-resource settings or when IVC imaging is suboptimal [32, 51].

## Discussion

Hyponatremia continues to challenge clinicians; however, this synthesis shows that bedside ultrasonography substantially reduces diagnostic uncertainty. A fundamental step in its proper management is the accurate assessment of extracellular volume status, which directs the differential diagnosis and guides therapy [10]. Traditionally, this assessment has relied on clinical examination and basic laboratory parameters, yet as we have noted, these tools can be insufficient or even misleading in a substantial proportion of cases. The classical signs of hypovolemia (dry mucous membranes, orthostasis, tachycardia, reduced skin turgor, oliguria, etc.) may not manifest—especially when hyponatremia is moderate or if the course is chronic and compensatory mechanisms have blunted the clinical picture [18]. We must also consider scenarios where euvolemic hyponatremia can masquerade as a hypovolemic or hypervolemic state. One notable example is advanced hypothyroidism (particularly myxedema). These patients exhibit generalized edema (due to mucopolysaccharide accumulation in subcutaneous tissue) and may develop ascites and pleural effusions. Such findings could mislead a clinician into categorizing the patient as hypervolemic, when the effective intravascular volume may be reduced [22]. Likewise, primary adrenal insufficiency (Addison’s disease) may initially present only with hyponatremia—often without the classic features—leading to repeated misdiagnoses of SIADH before the true cause is identified [36]. In general, an underlying adrenal or thyroid disorder should be considered in unexplained hyponatremia, even if typical signs, including hyperpigmentation or severe hypotension in Addison’s, are absent.

Taking all the above into account, one can appreciate the potential for inappropriate therapy that stems from misclassification. A patient with SIADH (euvolemic hyponatremia) who is erroneously thought to be hypovolemic may receive unnecessary isotonic saline—an intervention that not only fails to improve the hyponatremia but can worsen their condition by increasing free water retention. Conversely, a truly hypovolemic patient mistakenly diagnosed with SIADH might be managed only with fluid restriction, prolonging volume depletion and sustaining elevated ADH that perpetuates hyponatremia. Even in hypervolemic hyponatremia, underestimation of the degree of congestion may delay initiation of diuretics or vasopressin antagonists when indicated [19]. Our review underscores that integrating PoCUS into the assessment can mitigate these risks by improving diagnostic accuracy. In the prospective study by Rahman et al., the addition of ultrasound improved concordance with the final etiological diagnosis from ~ 63% to 84% (an absolute gain of over 20%) [37]—a clinically significant difference [46]. Moreover, recent diagnostic protocols, such as those outlined in the THAT study, have emphasized structured algorithms—including imaging—to prevent recurrent misdiagnoses in thiazide-associated hyponatremia [44].

It must be emphasized that PoCUS is a complement to, not a replacement for, the standard evaluation. A multi-modal approach remains essential: history, physical exam, and laboratory tests (plasma osmolality, urine osmolality and electrolytes, etc.) all provide critical pieces of information, and ultrasound findings should be interpreted in this holistic context. Used in this way, PoCUS becomes a powerful extension of our bedside assessment, resulting in a more nuanced, patient-specific evaluation. For example, if a patient is clinically suspected of SIADH (supported by symptoms and lab findings) but PoCUS shows clear signs of congestion, the clinician should reconsider that hypothesis—perhaps unmasking occult heart failure or renal impairment as contributors. *Similarly, in thiazide-induced hyponatremia, ultrasound findings may help differentiate true volume depletion from euvolemia or subclinical fluid overload, guiding more appropriate management decisions *[44]*.* This multi-dimensional perspective afforded by clinician-performed ultrasound aligns with calls to add “insonation” (ultrasound) as a fifth pillar of the bedside examination, alongside inspection, palpation, percussion, and auscultation. The end result is a form of precision medicine, where immediate ultrasound feedback allows tailored management decisions.

Several practical considerations about PoCUS merit discussion. First, ultrasound is operator-dependent—acquiring quality images and correctly interpreting subtle findings require adequate training and experience. Investment in training healthcare providers (via formal PoCUS courses and incorporation into residency curricula) is crucial. Encouragingly, many medical societies (including the Spanish Society of Internal Medicine) have begun integrating PoCUS training into their programs. Second, certain patient factors can limit ultrasound utility: morbid obesity, heavy chest wall musculature, or hyper-inflated lungs in COPD can all impede image acquisition. In such cases, interpretation must be cautious and alternative methods including bio-impedance for volume status, or invasive hemodynamic monitoring in critical care may be needed to guide management. Third, the overall evidence base for PoCUS in hyponatremia is still evolving. Up to October 2025, only one ongoing randomized trial (THAT Study) [44] has incorporated structured imaging parameters—including sonographic assessment of volume status—as co-variables to monitor response and refine phenotypic classification in patients with thiazide-associated hyponatremia. However, PoCUS is not the primary intervention under investigation. It remains to be demonstrated in prospective studies that a PoCUS-driven diagnostic and treatment approach improves hard outcomes (like osmotic demyelination rates, hospital length of stay, or mortality). Most existing data are observational, but the consistency of findings across independent reports lends credence to the recommendation for PoCUS use. One single-center study noted no significant difference in 24–96 h serum sodium correction between PoCUS-guided and standard assessments once appropriate therapy was administered [10]. This suggests that while PoCUS increases diagnostic confidence and may refine classification, it did not accelerate biochemical recovery in that cohort. Nonetheless, reducing diagnostic uncertainty and avoiding unnecessary or harmful interventions (as discussed above) are meaningful clinical benefits.

Crucially, PoCUS does not eliminate the need to investigate and treat the underlying causes of hyponatremia. Bedside ultrasound will not directly diagnose hypothyroidism or secondary adrenal insufficiency, although it might hint at them (for example, a pericardial effusion might raise suspicion of myxedema, or a lack of congestion might prompt checking adrenal function). Specific diagnostic tests (thyroid function, cortisol levels, etc.) remain indispensable when such etiologies are considered. In this sense, PoCUS augments but does not supplant the comprehensive workup; rather, it helps ensure that the workup is properly directed. This may confirm true euvolemia and thereby justifying an ADH evaluation, or revealing hidden hypovolemia and thus pointing to a volume-loss cause.

Bedside ultrasonography may enhance patient comprehension and involvement in clinical decisions. Performing a brief PoCUS exam (5–10 min) during the initial assessment can also save time and resources by avoiding diagnostic delays or incorrect empirical treatments [9, 19]. Mazón et al. (2024) have even developed a diagnostic algorithm that integrates PoCUS findings with conventional data for hyponatremia [20]. After confirming true hypotonic hyponatremia via lab tests, their protocol incorporates lung ultrasound, IVC measurement, VExUS grading, and focused cardiac ultrasound (including an estimate of cardiac output) to identify the likely volume status and cause, enabling tailored treatment. Although not yet widely validated, this personalized approach exemplifies how PoCUS can be systematically applied. One caveat is that measuring stroke volume and cardiac output with FoCUS requires additional training and often special software not available on all portable machines; however, these measurements can be learned using standard views and can yield valuable hemodynamic information in selected cases [26]. An additional limitation is that in prospective studies the final etiology was often adjudicated by clinical consensus rather than an independent gold standard, a methodological issue that may have led to overestimation of diagnostic accuracy.

Outside the specific context of hyponatremia, PoCUS-guided hemodynamic assessment has already shown clinical benefits in other contexts. For instance, in heart failure, ultrasound-guided decongestion strategies have been associated with more effective diuretic titration and improved outcomes, while in critical care, venous Doppler and fluid responsiveness assessments have refined fluid management decisions [48, 51]. These findings indirectly support the potential of PoCUS to enhance decision-making in hyponatremia, even though outcome benefits remain to be demonstrated specifically in this setting.

Future research should focus on larger, controlled trials to definitively determine whether PoCUS-guided management improves patient-centered outcomes in hyponatremia. Our review has some limitations as well: the available studies are relatively few and heterogeneous, precluding meta-analysis, and there may be publication bias (cases demonstrating PoCUS utility being more likely to be reported). Nevertheless, we aimed to include all relevant evidence up to October 2025 and provide a balanced view of strengths and limitations. The current body of evidence, while limited in scale, is remarkably consistent in demonstrating the advantages of PoCUS in this context [18, 22, 49–55].

## Conclusions

*PoCUS appears to improve the precision of volume status classification in hyponatremia*. Its multi-modal application—lung, cardiac, IVC, and VExUS—*may support identification of hypovolemia, euvolemia, and hypervolemia in ambiguous clinical settings* [4, 5, 13, 16, 23, 34, 37, 40, 45]. This can support more tailored management and help avoid therapeutic errors. PoCUS is also valuable for monitoring therapy and guiding dynamic interventions [5, 9, 12, 32, 37, 39, 41].

Given that current evidence is mainly descriptive, with only two comparative studies available and no proven outcome benefit, PoCUS should be regarded as a promising adjunct to the diagnostic workup of hyponatremia. Appropriate clinician training remains essential [20, 30, 49]. Further large, multicenter trials are required to determine its impact on clinical outcomes and cost-effectiveness [22, 36, 54]. Large, multicenter trials are needed to confirm outcome benefits and cost-effectiveness [26, 38, 56].

## Data Availability

Yale Tung Chen, principal investigator of the study, had full access to all data and takes responsibility for the integrity and the accuracy of the data analysis.
